# Association of Low Tumor Endothelial Cell pY397–Focal Adhesion Kinase Expression With Survival in Patients With Neoadjuvant-Treated Locally Advanced Breast Cancer

**DOI:** 10.1001/jamanetworkopen.2020.19304

**Published:** 2020-10-27

**Authors:** Marina Roy-Luzarraga, Tarek Abdel-Fatah, Louise E. Reynolds, Andrew Clear, Joseph G. Taylor, John G. Gribben, Stephen Chan, Louise Jones, Kairbaan Hodivala-Dilke

**Affiliations:** 1 Centre for Tumour Biology, Barts Cancer Institute, Queen Mary University of London, John Vane Science Centre, London, United Kingdom; 2 Department of Clinical Oncology, University of Nottingham and Nottingham University Hospitals NHS Trust, Nottingham, United Kingdom; 3 Pathology Department, National Liver Institute, Minoufyia University, Al Minufiyah, Egypt; 4 Centre for Haemato-Oncology, Barts Cancer Institute, Queen Mary University of London, John Vane Science Centre, London, United Kingdom

## Abstract

**Importance:**

Determining the risk of relapse after neoadjuvant chemotherapy in patients with locally advanced breast cancer is required to offer alternative therapeutic strategies.

**Objective:**

To examine whether endothelial cell phosphorylated–focal adhesion kinase (EC-pY397-FAK) expression in patients with treatment-naive locally advanced breast cancer is a biomarker for chemotherapy sensitivity and is associated with survival after neoadjuvant chemotherapy.

**Design, Setting, and Participants:**

In this prognostic study, expression levels of EC-pY397-FAK and tumor cell (TC)-pY397-FAK were determined by immunohistochemistry in prechemotherapy core biopsies from 82 female patients with locally advanced breast cancer treated with anthracycline-based combination neoadjuvant chemotherapy at Nottingham City Hospital in Nottingham, UK. Median follow-up time was 67 months. The study was conducted from December 1, 2010, to September 28, 2019, and data analysis was performed from October 2, 2019, to March 31, 2020.

**Exposures:**

All women underwent surgery followed by adjuvant radiotherapy and, if tumors were estrogen receptor–positive, 5-year tamoxifen treatment.

**Main Outcomes and Measures:**

Outcomes were pathologic complete response and 5-year relapse-free survival examined using Kaplan-Meier, univariable logistic, multivariable logistic, and Cox proportional hazards models.

**Results:**

A total of 82 women (age, 29-76 years) with locally advanced breast cancer (stage IIA-IIIC) were included. Of these, 21 women (26%) had high EC-pY397-FAK expression that was associated with estrogen receptor positivity (71% vs 46%; *P* = .04), progesterone receptor positivity (67% vs 39%; *P* = .03), high Ki67 (86% vs 41%; *P* < .001), 4-immunohistochemically stained luminal-B (52% vs 8%; *P* < .001), higher tumor category (T3/T4 category: 90% vs 59%; *P* = .01), high lymph node category (N2-3 category: 43% vs 5%; *P* < .001), and high tumor node metastasis stage (IIIA-IIIC: 90% vs 66%; *P* = .03). Of 21 patients with high EC-pY397-FAK expression levels, none showed pathologic complete response, compared with 11 of 61 patients with low EC-pY397-FAK expression levels who showed pathologic complete response (odds ratio, 0.70; 95% CI, 0.61-0.82; *P* = .04). High EC-pY397-FAK expression levels and high blood vessel density (BVD) were associated with shorter 5-year relapse-free survival compared with those with low EC-pY397-FAK expression levels (hazard ratio [HR], 2.21; 95% CI, 1.17-4.20; *P* = .01) and low BVD (HR, 2.2; 95% CI, 1.15-4.35; *P* = .02). High TC-pY397-FAK expression levels in 15 of 82 women (18%) were not associated significantly with pathologic complete response or 5-year relapse-free survival. A multivariable Cox regression model for 5-year relapse-free survival indicated that high EC-pY397-FAK expression levels was an independent poor prognostic factor after controlling for other validated prognostic factors (HR, 3.91; 95% CI, 1.42-10.74; *P* = .01). Combined analysis of EC-pY397-FAK expression levels, TC-pY397-FAK expression levels, and BVD improved prognostic significance over individually tested features.

**Conclusions and Relevance:**

The findings of this study suggest that low EC-pY397-FAK expression levels are associated with chemotherapy sensitivity and improved 5-year relapse-free survival after systemic therapy. Combined analysis of high EC-pY397-FAK expression levels, high TC-pY397-FAK expression levels, and high BVD appeared to identify a high-risk population.

## Key Points

**Question:**

Are phosphorylated–focal adhesion kinase (pY397-FAK) expression levels in endothelial or tumor cells and tumor blood vessel density associated with response to chemotherapy and relapse-free survival after neoadjuvant chemotherapy in patients with locally advanced breast cancer?

**Findings:**

In this prognostic study of 82 women with stage IIA to IIIC locally advanced breast cancer, low endothelial cell pY397-FAK expression levels in prechemotherapy core biopsies were associated with sensitivity to neoadjuvant chemotherapy and improved 5-year relapse-free survival. Combined analysis of high endothelial cell pY397-FAK, high tumor cell pY397-FAK, and high blood vessel density appeared to identify a high-risk population.

**Meaning:**

The findings of this study suggest that endothelial cell pY397-FAK levels could be used as a clinical tool for indicating response to neoadjuvant chemotherapy.

## Teaser

This prognostic study examines the use of endothelial cell phosphorylated-focal adhesion kinase as a biomarker for chemotherapy sensitivity in women with breast cancer.

## Introduction

Breast cancer is a heterogeneous disease and more than 2 million new cases of breast cancer are diagnosed worldwide each year in women.^1,2^ Despite the onset of new treatment strategies, mortality rates are estimated at approximately 600 000 women per year.^1^ There is a need to develop novel and effective biomarkers that could guide optimal management of the disease. Preoperative chemotherapy (neoadjuvant chemotherapy) is commonly given to patients with high-risk breast cancer, particularly triple-negative *ERBB2* (formerly *HER2*)-positive, and locally advanced breast cancer. Although chemotherapy reduces the early relapse rate (over the first 2 years) by 50%, the late relapse rate (8-year survival rate) is approximately 30%, and overall mortality is 20% to 25%.^3^ Current practice is largely based on assessment of the recurrence risk and overall survival of the patient, using the traditional clinicopathologic prognostic factors (eg, lymph node status) and the multigene tests (eg, Oncotype DX, Genomic Health; MammaPrint, Agendia; and Prosigna, Nanostring Technologies). Nevertheless, these tests do not determine whether a patient will respond to chemotherapy. Thus, developing biomarkers that will predict responses to chemotherapies is still required, and selecting those for which functional relevance to the biology of chemoresistance exists will likely provide more robust biomarkers.

Focal adhesion kinase (FAK) is a ubiquitously expressed nonreceptor tyrosine kinase that is upregulated in many carcinomas, including breast cancer.^4,5,6,7,8^ FAK activation results in FAK-tyrosine (Y)-397-phosphorylation.^9^ Bulk tumor FAK expression has been correlated with breast cancer progression, but definitive results vary.^6,10,11,12,13,14,15,16^ Evidence suggests that endothelial cell (EC) FAK expression levels correlate with the molecular subtype of breast cancer, indicating that EC-FAK expression is potentially more clinically relevant than tumor cell (TC) FAK expression in breast cancer.^10^

Deletion of FAK in K14-positive epidermal cells reduces skin cancer progression.^17^ FAK-kinase inactivation in the endothelium is associated with reduced vascular leakage,^18,19^ whereas the initiation of tumor angiogenesis is reduced by deletion of endothelial FAK in adult mice.^20^ In established tumors, loss of EC-FAK does not affect blood vessel density (BVD) but alters DNA damage-induced angiocrine signaling (eg, doxorubicin)^21,22,23^ and, in particular, reduces EC chemokine production. Reduced production of these angiocrine signals could govern chemosensitization in malignant cells, resulting in improved therapy efficacy in cancer control.^24^ Given that overexpression of FAK in many cancer types has led to the development of FAK inhibitors for cancer treatment, analysis of the association between endothelial FAK activation and clinical outcome after chemotherapy could be beneficial in selecting patients with breast cancer who may benefit from such novel therapy.

The purpose of this study was to examine whether phosphorylated (p)Y397-FAK expression levels in the endothelium of breast cancer before treatment are associated with response to anthracycline-based combination neoadjuvant chemotherapy and the subsequent risk of relapse.

## Methods

A cohort of 82 women with locally advanced breast cancer (categories T2-T4, N0-N3, and M0) from Nottingham University Hospital, Nottingham, UK, were included in a prognostic study conducted from December 1, 2010, to September 28, 2019. Analysis was begun October 2, 2019, and completed March 31, 2020. The study was approved by the institutional review board or independent ethics committee and the hospital research and innovations department at Nottingham University Hospital. Oral and written consent was obtained from participants before the investigation. Participants did not receive financial compensation. This study followed the Strengthening the Reporting of Observational Studies in Epidemiology (STROBE) reporting guideline for cohort studies.

### Measurement and Procedures

To quantify EC-pY397-FAK expression, core biopsies were sequentially immunohistochemically (IHC) stained for the endothelial cell marker, CD31, and for pY397-FAK, using a modified stripping and reprobing multiplex protocol based on previous reports^25,26,27^ (detailed protocol described in eMethods in the Supplement). Complete removal of antibody signal between pY397-FAK and CD31 staining was confirmed after stripping and when using control IgG nonspecific antibodies (eFigure 1A in the Supplement).

Expression of pY397-FAK in ECs and TCs of breast cancer sections was quantified (Visiopharm Quantitative Digital pathology software, Hoersholm). First, CD31-stained images were virtually aligned with FAK-stained images using the tissue align module. Correct alignment was manually checked for all samples. Damaged or nonaligned tissue areas were excluded from the analysis. To assess EC-pY397-FAK expression, CD31 staining was used to identify blood vessels and FAK intensity was measured only in this overlapping aligned area (eFigure 1B in the Supplement).

The median pY397-FAK intensity of all vessels per patient was used as the expression level (eTable 1 in the Supplement). Patients were classified into 2 groups (high and low) according to their EC-pY397-FAK median expression (eFigure 1C and D in the Supplement). The cutoff level of EC-pY397-FAK expression was determined by X-Tile software.^28^ Patients showed a similar coefficient of variation of EC-pY397-FAK expression levels (eFigure 2 in the Supplement).

To assess TC-pY397-FAK expression, 5 fields of epithelial tissue area per sample were manually selected and FAK expression levels were calculated using the same software. Mean TC-pY397-FAK expression levels were used per patient and X-Tile software was used to determine the cutoff value for patient classification. Blood vessel density per patient was calculated by dividing the vessel count by the tissue area analyzed. In this case, the median value was used as the cutoff level for the subsequent patient classification.

For validation of the method, intensity levels were also calculated using Image J software and were 1 and 1.5 times higher than those calculated with Visiopharm. There was no statistically significant difference between patients classified as high and low EC-pY397-FAK, indicating that both analyses could be used provided all samples were calculated using the same method (eFigure 3 in the Supplement).

### Outcome Measurements

The association of EC-pY397-FAK and TC-pY397-FAK protein expression and BVD levels in the diagnostic prechemotherapy core biopsy with clinicopathologic variables, pathologic complete response, and 5-year relapse-free survival were investigated. The clinicopathologic associations included estrogen receptor (ER), progesterone receptor (PR), *ERBB2*, Ki67, molecular phenotype determined by 4-IHC test, tumor T category, lymph node category, c-TNM stage, and histopathologic grade.

Pathologic complete response rate was defined as the absence of any neoplastic cells in both the primary breast site and lymph node after neoadjuvant chemotherapy. Five-year relapse-free survival was defined as the number of months from diagnosis to recurrence or distal metastasis relapse. Survival was censored if the patient was alive at the end of the study, lost to follow-up, or died from other causes.

### Statistical Analysis

Data analyses were performed using SPSS, version 17, software (SPSS Inc). Where appropriate, Pearson χ^2^ and *t* tests were used. Cumulative survival probabilities were estimated using the Kaplan-Meier method, and differences between survival rates were tested for significance using the log-rank test. Univariable analysis was used to evaluate baseline risk of 5-year risk of relapse, and both hazard ratios (HRs) and 95% CIs were calculated. Multivariable analyses for survival were performed using the Cox proportional hazards model after adjusting for other validated prognostic factors, such as ER, PR, *ERBB2*, and Ki67 expression, lymph node stage, T stage, grade, and pathologic complete response. The proportional hazards assumption was tested using standard log-log plots. Hazard ratios and 95% CIs were estimated for each variable.

For pathologic complete response analysis, univariable analysis as well as multivariable logistic regression tests were used and the odds ratio (OR) and 95% CI were calculated. All tests were 2-sided with a 95% CI, and a *P* < .05 was considered to indicate statistical significance.

## Results

The association between EC-pY397-FAK, TC-pY397-FAK protein expression levels, and BVD and clinicopathologic parameters, response to chemotherapy (pathologic complete response), and relapse-free survival were evaluated in the prechemotherapy core biopsies from 82 women (age, 29-76 years) with locally advanced breast cancer (stage IIA-IIIC), who were treated with anthracycline neoadjuvant chemotherapy at the Nottingham City Hospital. Details of the characteristics of the patient cohort are given in eTable 2 in the Supplement. Fifty-one percent (42/82) of the patients received 6 cycles of anthracycline-based therapy (ie, FEC [fluorouracil, 500 mg m^−2^, epirubicin, 75-100 mg m^−2^, and cyclophosphamide, 500 mg m^−2^, on day 1 of a 21-day cycle]), and 49% (40/82) of the patients received 3 cycles of the FEC regimen plus 3 cycles of docetaxel, 100 mg m^−2^. All patients underwent mastectomy or breast-conserving surgery and axillary dissection, followed by adjuvant radiotherapy and, if tumors were ER-positive (43/82 [52%]), 5-year tamoxifen treatment. For *ERBB2*-positive cancers (16/82 [20%]), adjuvant trastuzumab was prescribed for a year. The median follow-up time was 67 months (interquartile range, 27-81).

**Figure 1.**
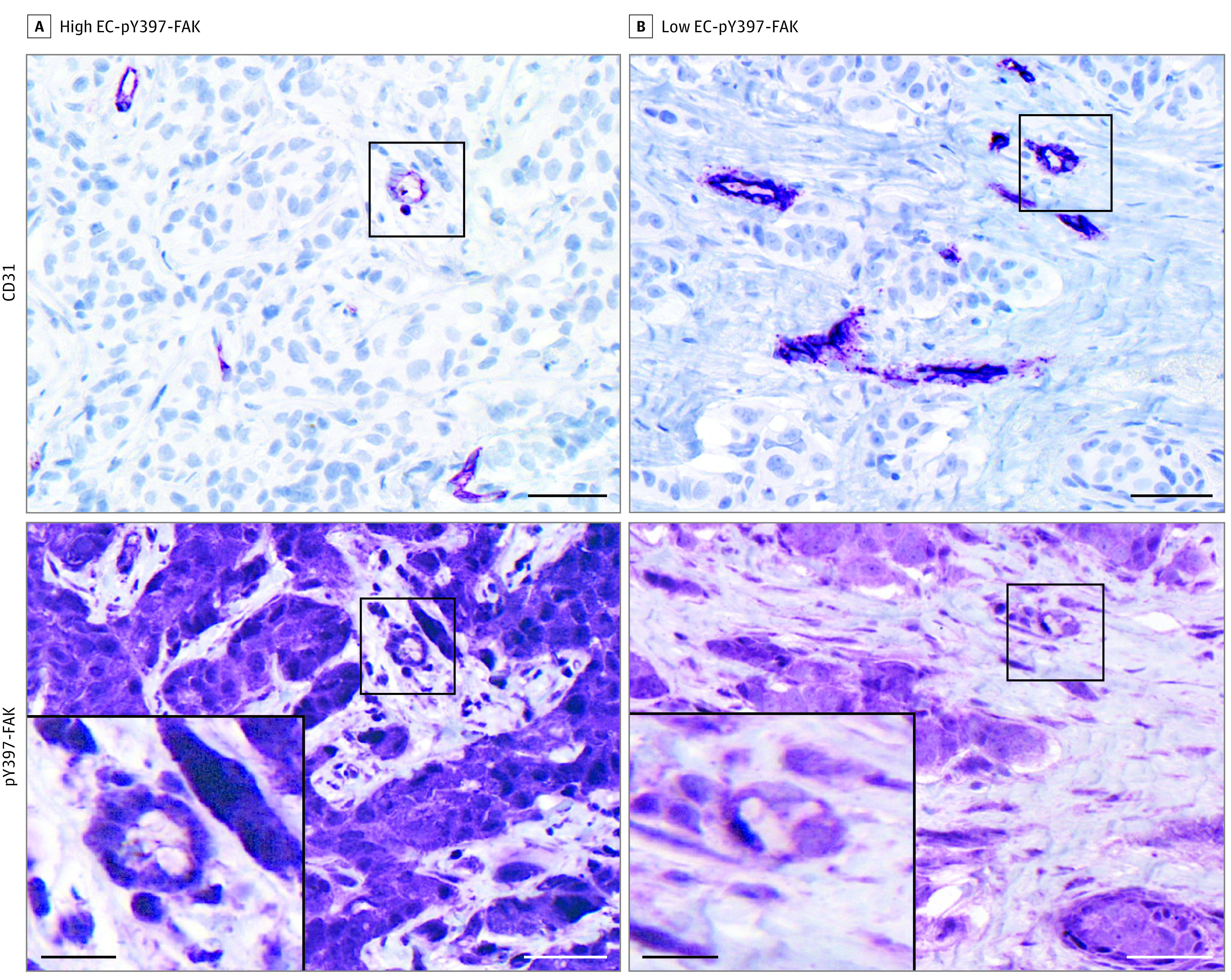
Immunohistochemical Analysis of High and Low Endothelial Cell Phosphorylated–Focal Adhesion Kinase (EC-pY397-FAK) Expression Levels Representative images of high (A) and low (B) EC-pY397-FAK staining. Sections were immunostained for CD31, stripped, and reprobed for pY397-FAK. Expression levels were measured digitally in pY397-FAK/CD31 overlapping areas. Scale bar, 50 μm. Higher magnification inserts, scale bar, 15 μm.

High and low EC-pY397-FAK expression levels were used to classify the 82 patients with locally advanced breast cancer (stage IIA-IIIC) in the study cohort (Figure 1). High EC-pY397-FAK expression levels (vessel median expression ≥121.96) were observed in 21 of 82 women (26%). Compared with low EC-pY397-FAK expression levels, high EC-pY397-FAK expression levels were associated with ER-positivity (71% vs 46%; *P* = .04), PR-positivity (67% vs 39%; *P* = .03), high Ki67 (86% vs 41%; *P* < .001), 4-IHC luminal-B (52% vs 8%; *P* < .001), higher T category (T3/T4: 90% vs 59%; *P* = .01), high lymph node category (N2-N3 category: 43% vs 5%; *P* < .001), and high TNM stage (IIIA-IIIC: 90% vs 66%, *P* = .03) (Table 1).

**Table 1.** Association Between EC-pY397-FAK Expression Levels and Clinicopathologic Factors

Variable	EC-pY397-FAK, No. (%)		*P* value^a^
	Low (n = 61)	High (n = 21)	
Tumor T category			
T1/T2 (≤5 cm)	25 (41)	2 (10)	.01
T3/T4 (>5 cm) or spread to chest wall and/or skin or inflammatory breast cancer	36 (59)	19 (90)	
Prechemotherapy lymph node stage (N category)			
N0	12 (20)	2 (10)	<.001
N1	46 (75)	10 (48)	
N2	0	8 (38)	
N3	3 (5)	1 (5)	
c-TNM stage			
IA-IIB	21 (34)	2 (10)	.03
IIIA-IIIC	40 (66)	19 (90)	
Histologic category			
Low (G1)/intermediate (G2)	30 (49)	12 (57)	.52
High (G3)	31 (51)	9 (43)	
Estrogen receptor			
Negative	33 (54)	6 (29)	.04
Positive	28 (46)	15 (71)	
Progesterone receptor			
Negative	37 (61)	7 (33)	.03
Positive	24 (39)	14 (67)	
*ERBB2* overexpression^b^			
Negative	47 (77)	19 (90)	.18
Positive	14 (23)	2 (10)	
Ki67 expression			
Low (<15%)	36 (59)	3 (14)	<.001
High (≥15%)	25 (41)	18 (86)	
Molecular phenotype			
Luminal-A (ER+/PR+/ *ERBB2*−/low Ki67)	17 (28)	3 (14)	.001
Luminal-B (ER+/PR+/ *ERBB2*−/high Ki67)	5 (8)	11 (52)	
Luminal *HER2* overexpression (ER+/*ERBB2*+)	7 (11)	1 (5)	
ER−/ *ERBB2*+	7 (11)	1 (5)	
ER−/ *ERBB2*−	25 (41)	5 (24)	
Pathologic complete response			
Yes	11 (18)	0	.04
No	50 (82)	21 (100)	

Abbreviations: EC-pY397-FAK, endothelial cell phosphorylated–focal adhesion kinase; ER, estrogen receptor; PR, progesterone receptor.

^a^ χ^2^ test.

^b^ *ERBB2*, formerly *HER2*.

**Figure 2.**
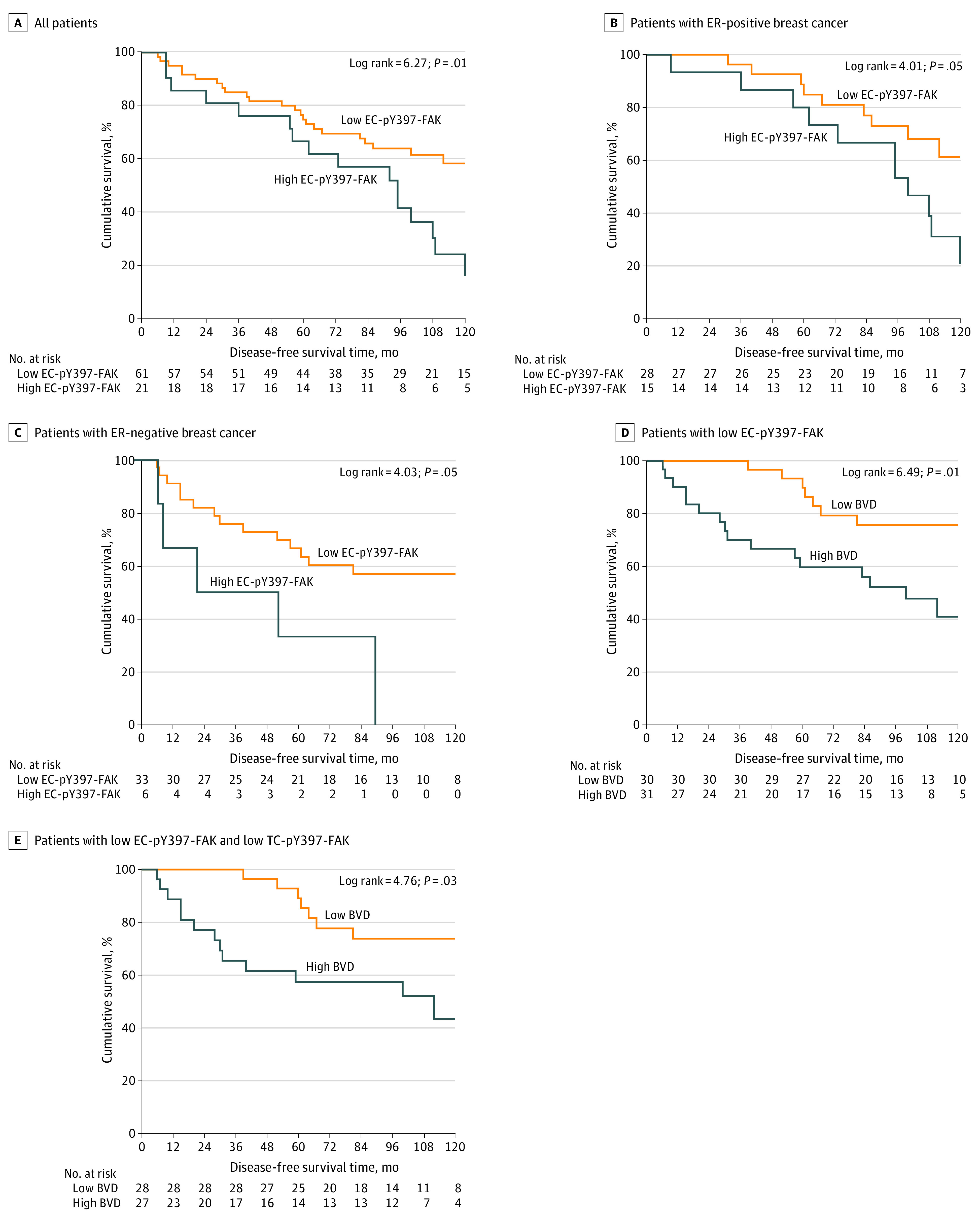
Endothelial Cell Phosphorylated–Focal Adhesion Kinase (EC-pY397-FAK) as a Prognostic Factor Rates of disease-free survival for low and high EC-pY397-FAK expression levels in all patients (A), estrogen receptor (ER)-positive patients (B), and ER-negative patients (C). (D) Disease-free survival rates in patients with low EC-pY397-FAK expression levels combined with low and high blood vessel density (BVD). (E) Disease-free survival rates of patients with low EC-pY397-FAK and low tumor cell (TC)-pY397-FAK expression levels combined with low and high BVD.

None of the patients with high EC-pY397-FAK expression levels showed pathologic complete response, whereas 11 of 61 patients with low EC-pY397-FAK expression levels showed pathologic complete response (0/21 vs 11/61; OR, 0.70; 95% CI, 0.61-0.82; *P* = .04) (Table 1). High EC-pY397-FAK expression levels were associated with shorter 5-year relapse-free survival compared with low EC-pY397-FAK expression levels (HR, 2.21; 95% CI, 1.17-4.20; *P* = .01) (Figure 2A). High EC-pY397-FAK expression levels were also associated with a significant reduction in 5-year relapse-free survival in the ER-positive subgroup, despite receiving neoadjuvant chemotherapy followed by 5 years of adjuvant endocrine therapy (HR, 2.4; 95% CI, 1.0-5.8; *P* = .05) (Figure 2B), and the ER-negative subgroup (HR, 2.9; 95% CI, 0.0-7.7; *P* = .05) (Figure 2C).

The number of blood vessels per unit area of tumor section was also measured to provide the BVD score. High tumor BVD was observed in 43 of 82 patients (52%) and, compared with low tumor BVD, was associated with 4-IHC luminal-B (28% vs 10%, *P* = .02), whereas low BVD compared with high BVD was associated with *ERBB2* overexpression (33% vs 7%, *P* = .003) (eTable 3 in the Supplement). High BVD was associated with shorter 5-year relapse-free survival (HR, 2.2; 95% CI, 1.15-4.35; *P* = .02) (eFigure 4 in the Supplement).

High TC-pY397-FAK expression levels (cutoff ≥127.91) were observed in 15 of 82 patients (18%) and, compared with low TC-pY397-FAK levels, were associated with ER-positivity (80% vs 46%; *P* = .01), 4-IHC luminal-B (67% vs 9%; *P* < .001), higher T category (T3/T4: 93% vs 61%; *P* = .01), and high TNM category (93% vs 67%, *P* = .04) (Table 2). Although an association between EC-pY397-FAK and TC-pY397-FAK expression was found (eTable 4 in the Supplement), no statistically significant associations between TC-pY397-FAK expression levels and pathologic complete response (*P* = .09) or TC-pY397-FAK expression levels and 5-year relapse-free survival were observed (Table 2; eFigure 5A in the Supplement). These results suggest that TC-pY397-FAK expression is not as relevant as EC-pY397-FAK expression levels in assessing prognosis. Furthermore, patients with low TC-pY397-FAK expression levels had longer 5-year relapse-free survival when they also had low EC-pY397-FAK expression levels compared with patients with low TC-pY397-FAK and high EC-pY397-FAK expression levels (eFigure 5B in the Supplement).

**Table 2.** Association Between TC-pY397-FAK Expression Levels and Clinicopathologic Factors

Variable	TC-pY397-FAK, No. (%)		*P* value^a^
	Low (n = 67)	High (n = 15)	
Tumor T category			
T1/T2 (≤5 cm)	26 (39)	1 (7)	.01
T3/T4 (>5 cm) or spread to chest wall and/or skin or inflammatory breast cancer	41 (61)	14 (93)	
Prechemotherapy lymph node stage (N category)			
N0	11 (16)	3 (20)	.94
N1	46 (69)	10 (67)	
N2	7 (10)	1 (7)	
N3	3 (4)	1 (7)	
c-TNM stage			
IA-IIB	22 (33)	1 (7)	.04
IIIA-IIIC	45 (67)	14 (93)	
Histologic grade			
Low (G1)/intermediate (G2)	33 (49)	9 (60)	.45
High (G3)	34 (51)	6 (40)	
Estrogen receptor			
Negative	36 (54)	3 (20)	.01
Positive	31 (46)	12 (80)	
Progesterone receptor			
Negative	39 (58)	5 (33)	.08
Positive	28 (42)	10 (67)	
*ERBB2* overexpression^b^			
Negative	54 (77)	12 (80)	.95
Positive	13 (32)	3 (20)	
Ki67 expression			
Low (<15%)	35 (52)	4 (27)	.07
High (≥15%)	32 (48)	11 (73)	
Molecular phenotype			
Luminal-A (ER+/PR+/ *ERBB2*−/low Ki67)	20 (30)	0	<.001
Luminal-B (ER+/PR+/ *ERBB2*−/high Ki67)	6 (9)	10 (67)	
Luminal *ERBB2* overexpression (ER+/*ERBB2*+)	6 (9)	2 (13)	
ER−/*ERBB2*+	7 (10)	1 (7)	
ER−/*ERBB2*−	28 (42)	2 (13)	
Pathologic complete response			
Yes	11 (16)	0	.09
No	56 (84)	15 (100)	

Abbreviations: ER, estrogen receptor; PR, progesterone receptor; TC-pY397-FAK, tumor cell phosphorylated–focal adhesion kinase.

^a^ χ^2^ test.

^b^ *ERBB2*, formerly *HER2*.

Combined analysis of patients with low EC-pY397-FAK and high BVD showed poorer clinical outcome than those with low EC-pY397-FAK and low BVD (Figure 2D). Combined analysis revealed that patients with low EC-pY397-FAK, low TC-pY397-FAK, and low BVD showed improved clinical outcome. Twenty-five patients (95%) survived without relapse at 5 years after treatment compared with patients with low EC-pY397-FAK, low TC-pY397-FAK, and high BVD (Figure 2E).

In a multivariable Cox proportional hazards model for 5-year relapse-free survival, high EC-pY397-FAK expression levels was an independent poor prognostic factor for relapse-free survival after adjusting for other validated prognostic factors, such as ER, PR, *ERBB2*, and Ki67 expression, lymph node stage, T category, and pathologic complete response (HR, 3.91; 95% CI, 1.42-10.74; *P* = .01). The interaction term between EC-pY397-FAK and TC-pY397-FAK expression was statistically significant. Also, the interaction between EC-pY397-FAK, TC-pY397-FAK, and BVD was statistically significant (Table 3).

**Table 3.** Multivariable Cox Proportional Hazards Analysis for 5-Year Disease-Free Survival

Variable	Hazard ratio (95% CI)	*P* value^a^
High EC-pY397-FAK expression	3.91 (1.42-10.74)	.01
High TC-pY397-FAK expression	0.30 (0.08-1.09)	.06
Blood vessel density	1.79 (0.81-3.97)	.15
High ER expression	1.15 (0.25-5.32)	.85
High PR expression	0.26 (0.05-1.28)	.09
*ERBB2* overexpression^b^	0.83 (0.28-4.47)	.20
High histologic grade	0.56 (0.27-1.18)	.12
High proliferation (Ki67)	1.13 (0.46-2.78)	.79
Pathologic complete response	0.34 (0.72-1.60)	.17
EC-pY397-FAK × TC-pY397-FAK expression (term interaction)	0.07 (0.01-0.47)	.01
BVD × TC-pY397-FAK expression (term interaction)	3.37 (0.92-12.34)	.07
EC-pY397-FAK × TC-pY397-FAK expression × BVD (term interaction)	0.18 (0.04-0.94)	.04

Abbreviations: BVD, blood vessel density; EC-pY397-FAK, endothelial cell phosphorylated–focal adhesion kinase; ER, estrogen receptor; PR, progesterone receptor; TC-pY397-FAK, tumor cell phosphorylated–focal adhesion kinase.

^a^ Cox proportional hazards model.

^b^ *ERBB2*, formerly *HER2*.

## Discussion

Biomarkers that inform appropriate treatment of breast cancer are part of the increase in success of breast cancer treatment.^29^ Despite this success, although anthracycline-based regimens have become standard of care, chemoresistance is still a major challenge.^30^ Thus, improved biomarkers are required to better inform and direct appropriate therapies, especially for high-risk patients. The role of the tumor microenvironment in the control of cancer progression is well established,^31^ but using a combination of molecular and morphologic tumor microenvironment–based biomarkers as prognostic indicators has been poorly explored. Our findings suggest that EC expression of activated FAK (EC-pY397-FAK) may be an independent prognostic biomarker for chemotherapy response in patients with advanced breast cancer who received neoadjuvant chemotherapy followed by adjuvant therapy. Combining biomarkers for EC-pY397-FAK, TC-pY397-FAK, and BVD may provide an improved relapse risk stratification over individual features alone. These data have potential therapeutic implications for high-risk populations that could benefit from additional novel therapy.

Several studies have linked high tumor FAK expression levels with poor prognosis in cancer,^6,9,16,32,33^ and others suggest that FAK expression does not correlate with outcome, showing no correlation with prognosis or that low FAK expression levels are associated with poor prognosis.^11,13,14,34,35,36,37^ For example, a study with 335 node-negative breast cancers indicated improved patient survival with high TC-FAK expression levels.^11^ The relevance of this finding to our study is limited since 83% of the cohort studied herein was node-positive. This apparent discrepancy highlights the idea that one biomarker may or may not be relevant in different subtypes of breast cancer and that understanding the biological functions of FAK in different breast cancer subtypes or at different stages of progression is of value.

The study of the potential role of FAK expression in predicting response to chemotherapy in human cancer is an emerging field,^16,38,39,40^ although most studies have not focused on FAK expression in blood vessels. A previous study described an association between EC-FAK expression levels and the molecular subtype of invasive breast cancer.^10^ Loss of EC-FAK is associated with improved outcome in patients with lymphoma after anthracycline-containing combination therapy, and loss of EC-FAK is sufficient to sensitize malignant cells to doxorubicin treatment in transgenic models of cancer.^24^ Further studies have shown that external signals, including growth factors,^8,18,41,42,43^ derived from the tumor microenvironment or tumor cells themselves induce activation of EC-pY397-FAK,^44^ and that FAK transduces ECM signals in a variety of different cell types,^45^ including ECs.^46,47,48,49^ Furthermore, an EC-Y397 nonphosphorylation mutation controls inside-out signaling, resulting in increased Tie-2 expression levels, reduced VEGFR-2 expression levels, decreased β_1_-integrin activation, and disrupted FAK/Src/PI3K(p55)/Akt signaling.^44^ These pY397-FAK–specific targets may be used as druggable targets for personalized therapies in patients who present with increased EC-pY397-FAK expression levels. For example, VEGFR-2 directed therapy is used in the clinic setting to target angiogenesis^50,51^; Src inhibitors,^52^ PI3K p55 specific inhibitors,^53^ and Akt inhibitors^54^ have all shown promising results in clinical trials.

Although previous reports have linked TC-FAK expression with lymphovascular invasion,^6^ we have seen this association only with high EC-pY397-FAK levels and not with TC-pY397-FAK levels. Our observations appear to corroborate preclinical studies in which EC-FAK regulates blood vessel permeability and metastasis.^19,42,55^ Given that high EC-pY397-FAK expression levels were associated not only with lymph node stage but also with relapse and that the main cause of relapse is metastatic spread, it is plausible that EC-pY397-FAK also contributes to breast cancer metastasis.

Previous studies have associated high BVD with poor prognosis,^56,57,58,59^ and the results from this cohort support these findings. Several clinical trials using antiangiogenics or vascular-disrupting agents alone or in combination with chemotherapy and endocrine therapy were conducted, but the therapy did not provide clinical benefit,^60^ possibly owing to failure in selecting subgroups of patients who would benefit from such therapies. Given that our data suggest that low EC-pY397-FAK expression levels with low BVD are associated with a better outcome than low EC-pY397-FAK expression levels with high BVD, future testing of vascular-disrupting agents in low EC-pY397-FAK expression levels with high BVD may provide added benefit and identify a group of patients who would benefit from targeting tumor blood vessels.

The upregulation of FAK in many carcinomas has led to the development of FAK inhibitors,^61,62^ and their benefits in breast cancer preclinical models have been demonstrated.^63,64,65^ Increasing evidence suggests a benefit of combining FAK-kinase inhibitors with chemotherapy in other types of cancer.^66,67,68,69^ Ongoing and future clinical trials will examine the value of FAK-kinase inhibitor treatment with chemotherapy in breast cancer progression and patient survival. Herein, however, our findings focused on the prognostic value of EC-pY397-FAK expression levels and its utility as a biomarker in identifying high- and low-risk patient subgroups to inform alternative treatment strategies.

This work could impact the management of therapy for patients with ER-positive breast cancer and may lead to the development of novel strategies for more effective management and treatment. For instance, high expression levels of EC-pY397-FAK and TC-pY397-FAK were more common in ER-positive luminal-B tumors and were associated with both chemotherapy and endocrine therapy resistance, providing an opportunity to select these patients for alternative novel therapies. Our findings have the potential to introduce an accurate predictive biomarker for identifying patients with ER-positive breast cancer who would or would not benefit from endocrine therapy and chemotherapy, and could also be used to monitor tumor response during treatment, thereby sparing patients with nonresponsive tumors the burden of adverse effects that would affect their quality of life. The next phase of our work is to develop this protocol into a diagnostic assay, which may prove to be cost-effective, and it will be validated in retrospective and prospective clinical studies and trials. This assay will help to deliver precision medicine for patient benefit.

Overall, our observations of poor prognosis in patients with breast cancer with high EC-pY397-FAK expression levels alone or in combination with high BVD open new opportunities for enriching recruitment studies of novel therapeutics for this population. Such novel therapeutics could be given instead of, concurrently with, or after standard of care treatment is completed. These high-risk patients could also benefit from more intensive follow-up. Further work will be required to streamline and accelerate the IHC and analysis processes for large numbers of patients. Given that protein expression is a dynamic and highly regulated process, it is possible that FAK has different roles at later stages of the disease. Thus, in future studies, it would also be of interest to determine the clinical relevance of EC-pY397-FAK in tumors taken at surgery.

### Limitations

This study had limitations. We used digital analysis to calculate EC-pY397-FAK expression levels. Although digital analysis avoids subjectivity and variability across pathologists, it only takes into consideration signal intensity without accounting for staining extent. Cutoff values will need to be standardized for future applications; however, the percentages of patients in each group used herein are comparable with those used in previously published work.^6,12^ The modest number of patients analyzed is also a potential limitation. To fully define the biological significance of EC-pY397-FAK expression levels as a marker of chemoresistance would require analysis in a series of patients with untreated disease, which is not feasible because withholding treatment would be unethical.

## Conclusions

The results of this study appear to support the significance of EC-pY397-FAK expression levels as an independent biomarker associated with advanced breast cancer therapy above TC-pY397-FAK expression or BVD. In addition, EC-pY397-FAK expression levels in combination with BVD and TC-pY397-FAK expression levels provide multiparametric biomarker associated with pathologic complete response. This study highlights the potential clinical utility of EC-pY397-FAK expression levels in combination with BVD to guide therapeutic decision-making and testing of novel therapeutics in the high-risk group. Our data also appear to support the usefulness of combining molecular and morphologic features in designing prognostic factors and therapy response.

## Notes

Supplement. **eMethods.** Detailed IHC Multiplex Experimental Protocol **eFigure 1.** Experimental Approach and Patient Classification by EC-pY397-FAK Expression **eTable 1.** Median EC-pY397-FAK Expression Values per Individual Patient **eFigure 2.** Variation of EC-pY397-FAK Expression Patterns Among Patients **eFigure 3.** Comparison of Image J With Visiopharm Software for EC-pY397-FAK Expression Quantification **eTable 2.** Clinical, Pathological and Molecular Features of the NUH Locally Advanced Breast Cancer Patient Cohort **eTable 3.** Association Between BVD and Clinicopathological Factors **eFigure 4.** High BVD Is Associated With Poor Prognosis **eTable 4.** Association Between EC-pY397-FAK Expression and TC-pY397-FAK Expression or Blood Vessel Density (BVD) **eFigure 5.** TC-pY397-FAK Expression Alone Is Not Associated With Clinical Outcome but Combination of Low TC-pY397-FAK Expression With Low EC-pY397-FAK Expression Has Prognostic Value
