# Supplementary material for: Association of Low Tumor Endothelial Cell pY397–Focal Adhesion Kinase Expression With Survival in Patients With Neoadjuvant-Treated Locally Advanced Breast Cancer
Source: JAMA Netw Open. 2020 Oct 27;3(10):e2019304. doi: 10.1001/jamanetworkopen.2020.19304 (PMC7592032; doi:10.1001/jamanetworkopen.2020.19304)
Supplement: Supplement. — eMethods. Detailed IHC Multiplex Experimental Protocol eFigure 1. Experimental Approach and Patient Classification by EC-pY397-FAK Expression eTable 1. Median EC-pY397-FAK Expression Values per Individual Patient eFigure 2. Variation of EC-pY397-FAK Expression Patterns Among Patients eFigure 3. Comparison of Image J With Visiopharm Software for EC-pY397-FAK Expression Quantification eTable 2. Clinical, Pathological and Molecular Features of the NUH Locally Advanced Breast Cancer Patient Cohort eTable 3. Association Between BVD and Clinicopathological Factors eFigure 4. High BVD Is Associated With Poor Prognosis eTable 4. Association Between EC-pY397-FAK Expression and TC-pY397-FAK Expression or Blood Vessel Density (BVD) eFigure 5. TC-pY397-FAK Expression Alone Is Not Associated With Clinical Outcome but Combination of Low TC-pY397-FAK Expression With Low EC-pY397-FAK Expression Has Prognostic Value [file jamanetwopen-e2019304-s001.pdf]

Supplementary Online Content

Roy-Luzarraga M, Abdel-Fatah T, Reynolds LE, et al. Association of low tumor endothelial cell pY397-focal adhesion kinase expression with survival in patients with neoadjuvant-treated locally advanced breast cancer. *JAMA Netw Open*. 2020;3(10):e2019304. doi:10.1001/jamanetworkopen.2020.19304

eMethods. Detailed IHC Multiplex Experimental Protocol

eFigure 1. Experimental Approach and Patient Classification by EC-pY397-FAK Expression

eTable 1. Median EC-pY397-FAK Expression Values per Individual Patient

eFigure 2. Variation of EC-pY397-FAK Expression Patterns Among Patients

eFigure 3. Comparison of Image J With Visiopharm Software for EC-pY397-FAK Expression Quantification

eTable 2. Clinical, Pathological and Molecular Features of the NUH Locally Advanced Breast Cancer Patient Cohort

eTable 3. Association Between BVD and Clinicopathological Factors

eFigure 4. High BVD Is Associated With Poor Prognosis

eTable 4. Association Between EC-pY397-FAK Expression and TC-pY397-FAK Expression or Blood Vessel Density (BVD)

eFigure 5. TC-pY397-FAK Expression Alone Is Not Associated With Clinical Outcome but Combination of Low TC-pY397-FAK Expression With Low EC-pY397-FAK Expression Has Prognostic Value

This supplementary material has been provided by the authors to give readers additional information about their work.

© 2020 Roy-Luzarraga M et al. *JAMA Network Open.*

eMethods. Detailed IHC multiplex experimental protocol.

Stripping and re-probing multiplex protocol

Sections were deparaffinised in xylene and rehydrated through a series of ethanols to water. Endogenous peroxidase was blocked with 3% hydrogen peroxide. Antigen retrieval was performed with a commercial citrate-based unmasking buffer (Vector Labs) at 120ºC using a pressure cooker. Sections were incubated for 40 minutes with CD31 (Dako M0823, 1:2000). Detection of primary antibody was performed using the Super-sensitive – Polymer HRP system (Biogenex) and staining was visualized using the purple chromogen VIP (Vector Labs). Slides were scanned (Panoramic 250 Flash) then left to soak in xylene to de-coverslip. Slides were rehydrated through ethanol to water. De-staining was achieved using a subsequent round of antigen retrieval, as above, followed by incubation with the second primary antibody pY397-FAK (Invitrogen 700255, 1:750), then detection, visualization and scanning. IgG negative controls showed specificity of antibodies.

© 2020 Roy-Luzarraga M et al. *JAMA Network Open.*

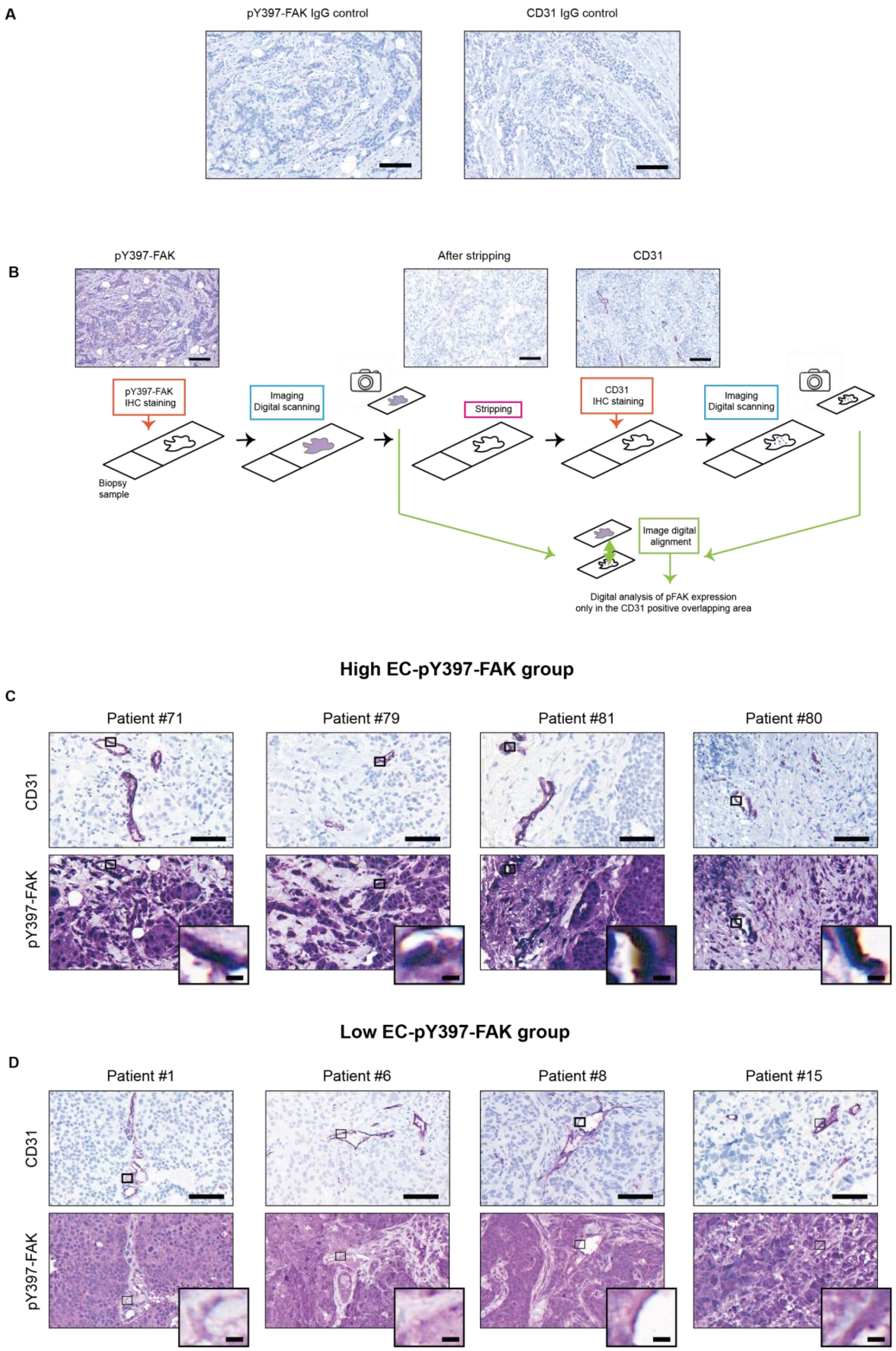

eFigure 1. **Experimental approach and patient classification by EC-pY397-FAK expression.** (**A**) Controls for immunohistochemical analysis: IgG controls for pY397-FAK antibody (left) and CD31 antibody (right); scale bar 100 µm. (**B**) Pipeline of the experimental design including images of p-Y397-FAK staining (left), after stripping (centre) and CD31 staining (right); scale bar 100 µm. Representative images of CD31 and p-Y397-FAK staining of patients classified with (**C**) high and (**D**) low EC-pY397-FAK expression. All stainings were visualized using the purple chromogen VIP; scale bar 65 µm. High magnification inserts of pY397-FAK expression in blood vessels are given; scale bar 5 µm.

© 2020 Roy-Luzarraga M et al. *JAMA Network Open.*

eTable 1. Median EC-pY397-FAK expression values per individual patient.

Patient number	Median EC-pY397-FAK expression
Patient #1	63.94
Patient #2	65.47
Patient #3	65.99
Patient #4	66.92
Patient #5	74.95
Patient #6	76.30
Patient #7	79.96
Patient #8	79.99
Patient #9	82.99
Patient #10	83.43
Patient #11	84.98
Patient #12	85.94
Patient #13	85.97
Patient #14	88.96
Patient #15	89.46
Patient #16	89.97
Patient #17	90.42
Patient #18	90.62
Patient #19	90.99
Patient #20	92.43
Patient #21	92.95
Patient #22	93.48
Patient #23	95.94
Patient #24	95.98
Patient #25	95.99
Patient #26	95.99
Patient #27	96.46
Patient #28	96.93
Patient #29	100.45
Patient #30	101.65
Patient #31	102.29
Patient #32	103.72
Patient #33	104.30
Patient #34	104.63
Patient #35	104.96
Patient #36	106.46
Patient #37	106.95
Patient #38	106.96
Patient #39	106.99
Patient #40	107.95
Patient #41	108.45
Patient #42	108.97
Patient #43	108.98
Patient #44	109.94

© 2020 Roy-Luzarraga M et al. *JAMA Network Open.*

Patient number	**Median EC-pY397-FAK expression**
Patient #45	109.95
Patient #46	110.24
Patient #47	110.97
Patient #48	110.97
Patient #49	111.97
Patient #50	114.21
Patient #51	114.21
Patient #52	114.99
Patient #53	115.95
Patient #54	115.98
Patient #55	116.45
Patient #56	116.98
Patient #57	117.72
Patient #58	119.31
Patient #59	120.96
Patient #60	120.96
Patient #61	121.90
Patient #62	121.96
Patient #63	123.62
Patient #64	124.98
Patient #65	127.72
Patient #66	129.46
Patient #67	129.46
Patient #68	129.96
Patient #69	129.99
Patient #70	131.98
Patient #71	132.31
Patient #72	133.48
Patient #73	133.97
Patient #74	136.17
Patient #75	137.77
Patient #76	137.96
Patient #77	137.96
Patient #78	138.97
Patient #79	140.95
Patient #80	144.21
Patient #81	144.96
Patient #82	153.65

© 2020 Roy-Luzarraga M et al. *JAMA Network Open.*

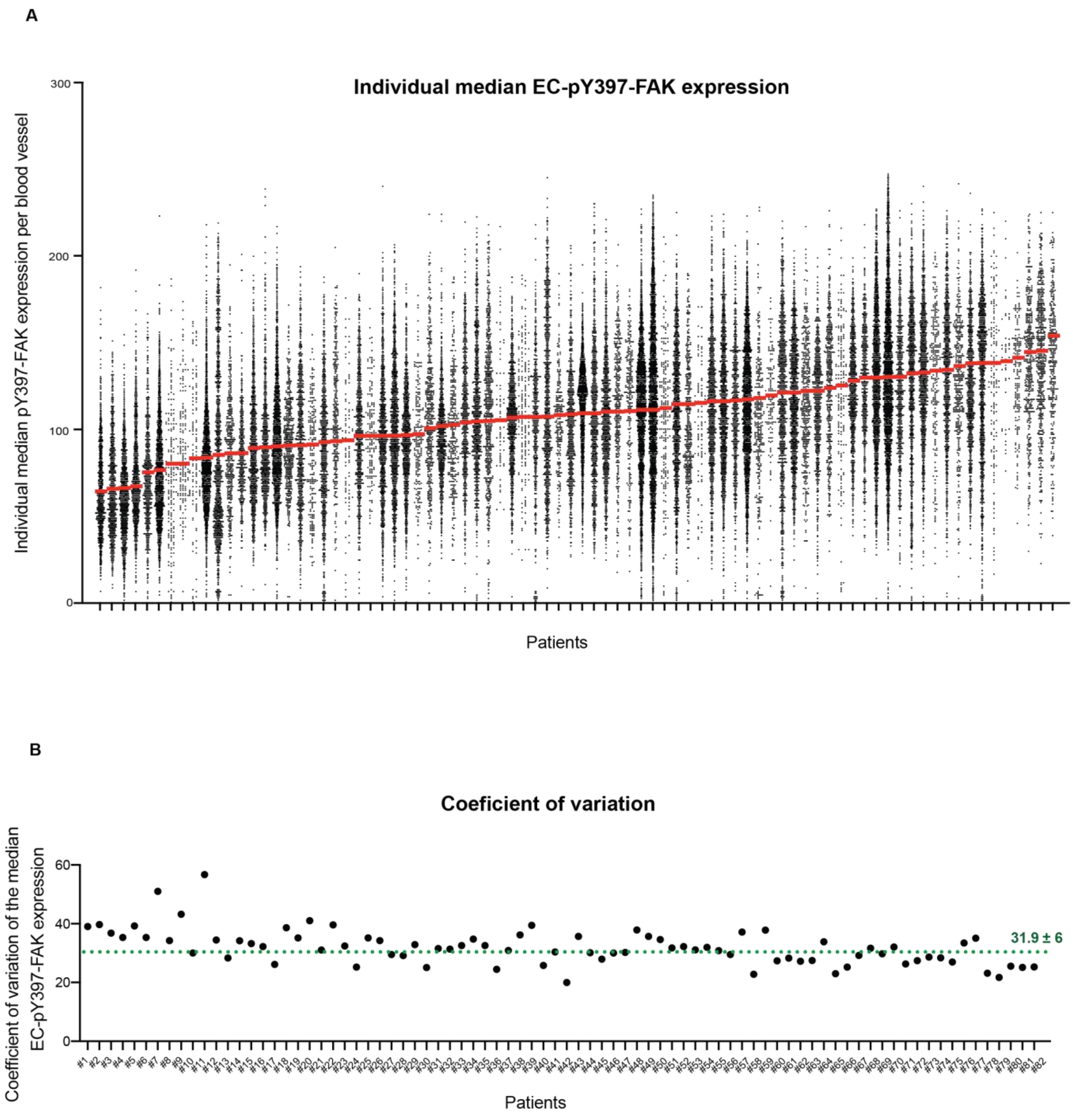

eFigure 2. **Variation of EC-pY397-FAK expression patterns among patients.** (**A**) Scatter plot of individual median EC-pY397-FAK expression values per blood vessel for all patients. Red lines indicate median EC-pY397-FAK expression per patient. (**B**) Coefficient of variation (%) among EC- pY397-FAK median expression per patient. Green dotted line shows mean of coefficient of variation.

© 2020 Roy-Luzarraga M et al. *JAMA Network Open.*

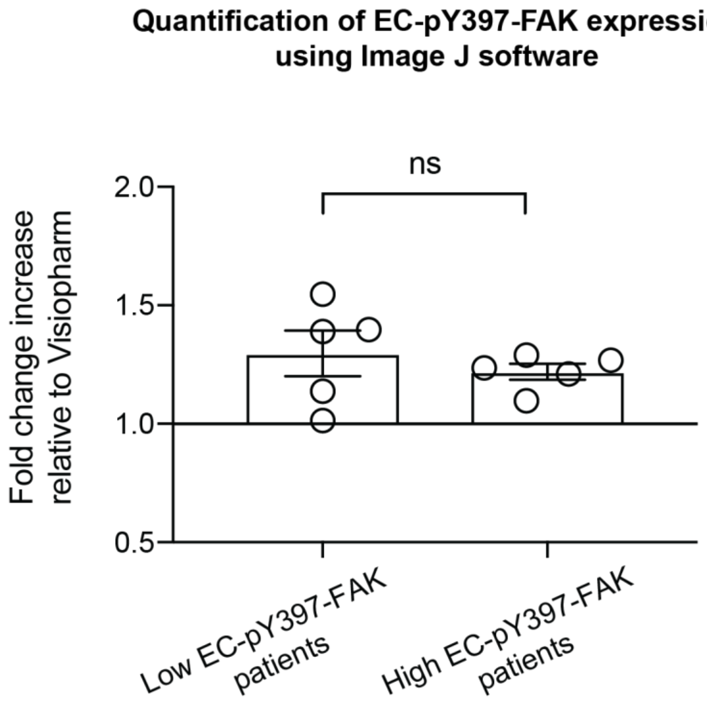

eFigure 3. **Comparison of Image J with Visiopharm software for EC-pY397-FAK expression quantification.** EC- pY397-FAK expression of 5 patients classified as high and low EC- pY397-FAK, were calculated using Visiopharm and ImageJ software. Fold change increase between Image J mean expression and Visiopharm are given per patient. Bar charts show mean values ± SEM; t-test applied; ns: non-statistically significant.

© 2020 Roy-Luzarraga M et al. *JAMA Network Open.*

eTable 2. Clinical, pathological and molecular features of the NUH locally advanced breast cancer patient cohort (n=82).

Characteristic		Number of patients	Percentage
Tumor T stage	T1 (≤2 cm)	4	5 %
	T2 ((≤5 cm)	20	24 %
	T3 (>5 cm)	19	23 %
	T4 (Inflammatory breast cancer)	39	48 %
Pre-Chemotherapy Lymph node Stage (N stage)	N0	14	17 %
	N1	56	68 %
	N2	8	10 %
	N3	4	5 %
c-TNM	IA-IIA	7	8 %
	IIB	16	20 %
	IIIA	16	20 %
	IIIB	38	46 %
	IIIC	5	6 %
Histological Grade	Low (G1) /Intermediate (G2)	42	51 %
	High (G3)	40	49 %
Histological type	IDC	69	84 %
	ILC	11	13 %
	Others	2	3 %
Estrogen receptor	Negative	39	48 %
	Positive	43	52 %
Progesterone receptor	Negative	44	54 %
	Positive	38	46 %
ERBB2 overexpression	Yes	16	20 %
	No	66	80 %
Ki67 expression	Low (<15%)	39	48 %
	High (>=15&	43	52 %
Molecular phenotype (4 IHC)	Luminal A (ER+/PR+/ *ERBB2*-/ low proliferation	20	24 %
	Luminal B (ER+/PR+/ *ERBB2*-/high proliferation	16	19 %
	Luminal *ERBB2+*	8	10 %
	ER-/ *ERBB2+*	8	10 %
	ER-/ *ERBB2-*	30	37 %
Pathologic complete response (pCR)	Yes	11	13 %
	No	71	87 %
5-year relapse	No	43	52 %
	Yes	39	48 %
Pre-operative chemotherapy regimen	Anthracycline based combination (FAC/FEC)	42	51 %
	Anthracycline based combination + Taxane (FEC-T)	40	49 %
Adjuvant Radiation therapy	Yes	82	100%
	No	0	0%
Adjuvant Endocrine therapy	Yes	43	52 %
	No	39	48 %
Trastuzumab treatment	Yes	16	20 %
	No	66	80 %

© 2020 Roy-Luzarraga M et al. *JAMA Network Open.*

eTable 3. Association between BVD and clinicopathological factors.

Variables	Low BVD n=39	High BVD n=43	P value
Tumor T stage<br>T1/T2 (=≤5 cm)<br>T3/T4 (>5 cm) or spread to chest wall and or skin or inflammatory breast cancer	14 (36%)<br>25 (64%)	13 (30%)<br>30 (70%)	0.58
Pre-Chemotherapy Lymph node Stage (N stage)<br>N0<br>N1<br>N2<br>N3	10 (25%)<br>23 (59%)<br>3 (8%)<br>3 (8%)	4 (9%)<br>33 (77%)<br>5 (12%)<br>1 (2%)	0.12
c-TNM stage<br>IA-IIB<br>IIIA-IIIC	12 (31%)<br>27 (69%)	11 (26%)<br>14 (74%)	0.60
Histological Grade<br>Low (G1)/Intermediate (G2)<br>High (G3)	21 (54%)<br>18 (46%)	21 (49%)<br>22 (51%)	0.65
Estrogen receptor (ER)<br>Negative<br>Positive	19 (49%)<br>20 (51%)	20 (46%)<br>23 (54%)	0.84
Progesterone receptor (PR)<br>Negative<br>Positive	21 (54%)<br>18 (46%)	23 (54%)<br>20 (46%)	0.97
ERBB2 overexpression<br>Negative<br>Positive	26 (67%)<br>13 (33%)	40 (93%)<br>3 (7%)	0.003*
Ki67 expression<br>Low (<15%)<br>High (>=15%)	20 (51%)<br>19 (49%)	19 (44%)<br>24 (56%)	0.52
Molecular phenotype<br>Luminal A (ER+/PR+/ *ERBB2*-/ low Ki67)<br>Luminal B (ER+/PR+/ *ERBB2*-/high Ki67)<br>Luminal HER2 overexp. (ER+ *ERBB2*+)<br>ER-/ *ERBB2*+<br>ER-/ *ERBB2*-	10 (26%)<br>4 (10%)<br>7 (18%)<br>6 (15%)<br>12 (31%)	10 (23%)<br>12 (28%)<br>1 (2%)<br>2 (5%)<br>18 (42%)	0.02*
Pathologic complete response (pCR)<br>Yes<br>No	7 (18%)<br>32 (82%)	4 (9%)<br>39 (91%)	0.25

*P<0.05; Chi-square test.

© 2020 Roy-Luzarraga M et al. *JAMA Network Open.*

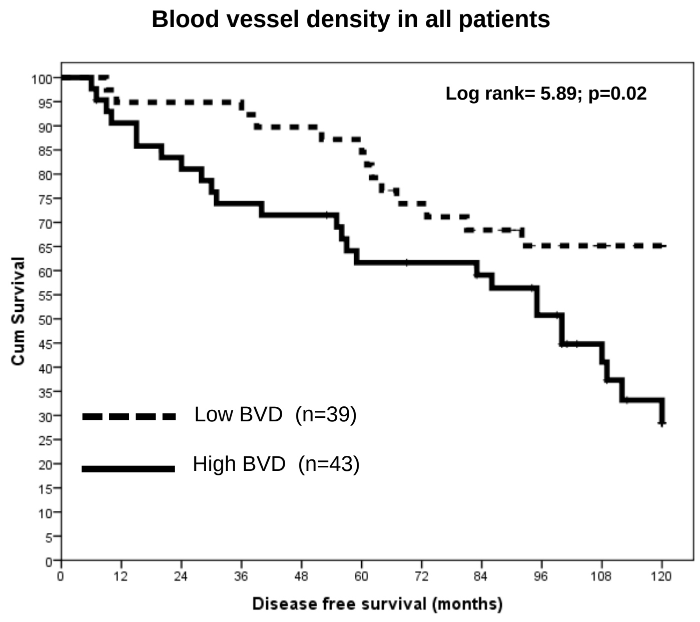

eFigure 4. High BVD is associated with poor prognosis. Kaplan-Meier plot of the rates of disease-free survival (DFS) according to BVD of all patients. P-value from Log rank test is shown. n: number of cases per group as indicated.

© 2020 Roy-Luzarraga M et al. *JAMA Network Open.*

eTable 4. Association between EC-pY397-FAK expression and TC-pY397-FAK expression or Blood vessel density (BVD).

Variables	Low EC-pY397-FAK n=61	High EC-pY397-FAK n=21	P value
Tumor cell-pY397-FAK			0.001*
Low	55 (90%)	12 (57%)	
High	6 (10%)	9 (43%)	
Blood vessels density (BVD)			0.09
Low	49 (80%)	13 (62%)	
High	12 (20%)	8 (38%)	

*P<0.05; Chi-square test.

© 2020 Roy-Luzarraga M et al. *JAMA Network Open.*

A

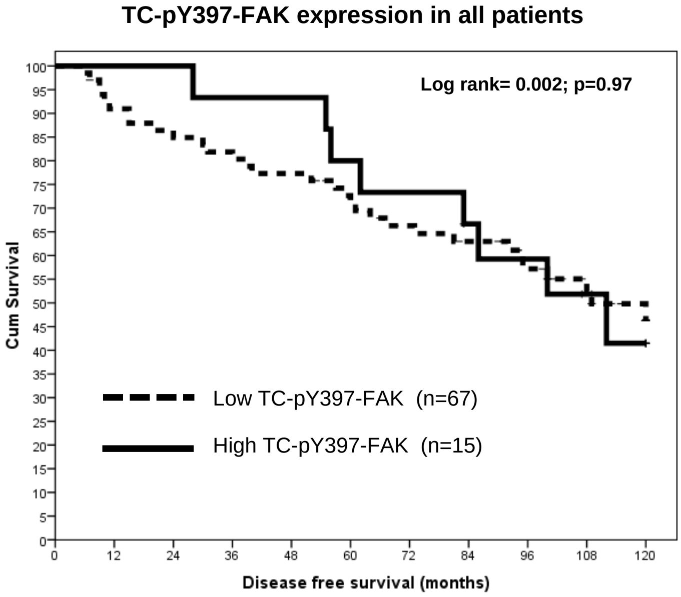

B

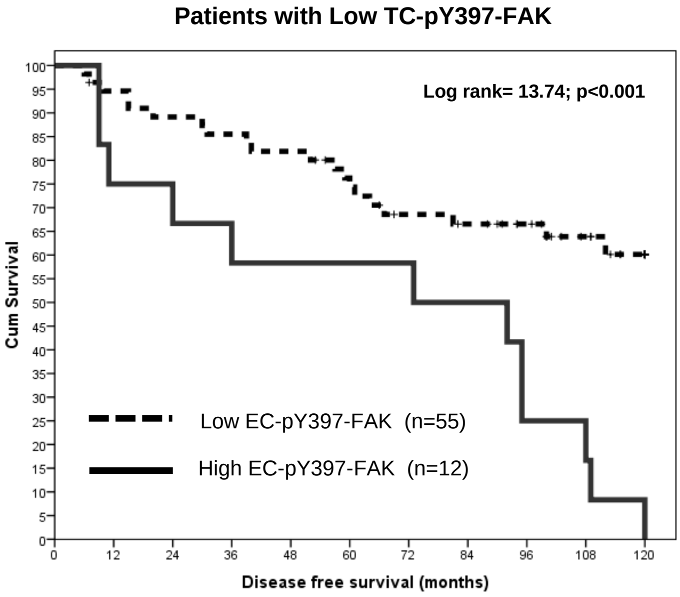

eFigure 5. TC-pY397-FAK expression alone is not associated with clinical outcome but combination of low TC-pY397-FAK expression with low EC-pY397-FAK expression has prognostic value. (**A**) Kaplan-Meier plots of DFS rates according to low and high TC-pY397-FAK expression of all patients. (**B**) Kaplan-Meier plots of DFS rates of patients with combined low TC-pY397-FAK and either low or high EC-pY397-FAK expression. P-value from Log rank test is shown in each panel. n: number of cases per group as indicated.

© 2020 Roy-Luzarraga M et al. *JAMA Network Open.*
